# Leakage-Flow Models for Screw Extruders

**DOI:** 10.3390/polym13121919

**Published:** 2021-06-09

**Authors:** Christian Marschik, Wolfgang Roland, Marius Dörner, Georg Steinbichler, Volker Schöppner

**Affiliations:** 1 Institute of Polymer Extrusion and Compounding, Johannes Kepler University Linz, 4040 Linz, Austria; wolfgang.roland@jku.at (W.R.); georg.steinbichler@jku.at (G.S.); 2 Competence Center CHASE GmbH, 4040 Linz, Austria; 3 Kunststofftechnik Paderborn, Paderborn University, 33098 Paderborn, Germany; marius.doerner@ktp.uni-paderborn.de (M.D.); Volker.Schoeppner@ktp.upb.de (V.S.)

**Keywords:** leakage flow, extrusion, modeling and simulation, polymer processing

## Abstract

Many theoretical analyses of extrusion ignore the effect of the flight clearance when predicting the pumping capability of a screw. This might be reasonable for conventional extruder screws with “normal” clearances but leads to errors when more advanced screw designs are considered. We present new leakage-flow models that allow the effect of the flight clearance to be included in the analysis of melt-conveying zones. Rather than directly correcting the drag and pressure flows, we derived regression models to predict locally the shear-thinning flow through the flight clearance. Using a hybrid modeling approach that includes analytical, numerical, and data-based modeling techniques enabled us to construct fast and accurate regressions for calculating flow rate and dissipation rate in the leakage gap. Using the novel regression models in combination with network theory, the new approximations consider the effect of the flight clearance in the predictions of pumping capability, power consumption and temperature development without modifying the equations for the down-channel flow. Unlike other approaches, our method is not limited to any specific screw designs or processing conditions.

## 1. Introduction

Plasticating extruders abound in the polymer industry. Due to their great versatility, they are used in many polymer-shaping operations, producing semi-finished plastic products such as films, pipes, profiles, sheets, and fibers. In addition, they are frequently found in compounding and recycling operations. Although plasticating extruders come in various designs, the elementary processing steps are generally the same: (i) transport and (ii) melting of particulate solids, followed by (iii) mixing and (iv) pumping of the polymer melt.

Numerous theoretical studies have modeled the extrusion process to increase the understanding of the transport mechanisms governing physical operation. The fundamentals of extrusion modeling were summarized in various books: Tadmor and Klein [1], White and Potente [2], Tadmor and Gogos [3], Campbell and Spalding [4], Rauwendaal [5], Agassant et al. [6], and others. Recently, Wilczyński et al. [7] presented a thorough review of global extrusion modeling.

The assessment of leakage flow and its prediction has been of interest since the earliest theories for the metering zone. Over the past decades, many analyses have modeled polymer-melt flows in single-screw extruders. Most of these, however, ignored the effect of flight clearance.

### 1.1. Analysis of Flow in Metering Channels

Early theoretical analyses that investigated melt conveying in single-screw extruders dealt with one- and two-dimensional flows of temperature-independent Newtonian fluids. These problems have exact analytical solutions for the drag and pressure flows in the cross- and down-channel directions, respectively. The first model of screw viscosity pumps was published anonymously [8] and later extended by Rowell and Finlayson [9]. Similarly, Carley et al. [10] proposed a simplified flow theory for screw extruders. Mohr et al. [11,12] investigated the characteristics of the transverse flow in the screw channel. Both the complexity and the accuracy of the analysis increase when the non-Newtonian flow behavior of polymer melts is included. It is well known that polymer melts are shear-thinning fluids whose viscosity decreases with increasing shear rate. Pseudo-plastic behavior complicates the mathematical model such that the governing flow equations must be solved numerically, and exact closed-form analytical solutions are no longer possible. The viscosity being dependent on the shear rate, the drag and pressure flows are coupled. For multidimensional flows, complexity is increased further by the combined effect of shear in the down- and cross-channel directions. To gain insights into how a material’s shear-thinning nature affects melt conveying in single-screw extruders, several authors presented numerical solutions for one- and two-dimensional flows of power-law fluids in metering channels. Rotem and Shinnar [13] obtained numerical results for a one-dimensional flow under isothermal conditions. Including the effect of transverse flow, Griffith [14], Zamodits and Pearson [15], and Karwe and Jaluria [16] presented numerical solutions for shear-thinning flows in infinitely-wide screw channels.

With the development of more advanced computers, a trend emerged towards a numerical analysis of three-dimensional flows in metering channels. Including non-Newtonian and non-isothermal effects, a number of studies provided detailed insights into the recirculating extruder channel flow. Spalding et al. [17] applied the finite-element method to examine the flow in a helical metering section. Ghoreishy et al. [18] presented a non-isothermal analysis of elastomer melt flow in unwound screw channels. Polychronopoulos and Vlachopoulos [19] analyzed the development of Moffat Eddies in the flight root corners of the screw channel. Vachagina et al. [20] investigated the non-isothermal conveying behavior of a Giesekus fluid in a helical screw section.

To avoid numerical procedures, various approximate solutions for shear-thinning flows were developed. Booy [21] employed the classical Newtonian pumping model to derive effective viscosity values and approximate shear-thinning flow behavior. Rau-wendaal [22] proposed correction factors to the drag- and pressure flows in the tra-ditional model to include the shear-rate-dependent flow behavior of polymer melts. Similarly, Spalding et al. [23] introduced a correction factor for the drag flow.

Rather than presenting corrections to the traditional Newtonian theory, we pro-posed an alternative approach for power-law fluids. Using a hybrid modeling approach that combines analytical, numerical, and data-based modeling techniques, we approximated a large number of numerical solutions of scaled flow equations obtained for two- and three-dimensional formulations. Assuming two-dimensional flows of power-law fluids in infinitely-wide screw channels, Pachner et al. [24] and Roland et al. [25,26] proposed regression models for the prediction of throughput and viscous dissipation. To include the influence of the flight flanks, we [27,28,29,30] extended the theories to three-dimensional flows. These widely applicable models increase prediction accuracy by including the combined effects of shear-thinning flow behavior, transverse flow, and screw flights.

### 1.2. Analysis of Leakage Flow

The influence of leakage flow on the pumping capability of extruder screws has been investigated since the earliest theories for the metering zone. Rowell and Finlayson [9] approximated the annular clearance between flight land and barrel surface by two parallel plates and assumed the leakage flow to be a pressure flow through an infinitely wide slit. Gore and McKelvey [31] extended the analysis by adding the effect of flight clearance on the drag flow. Mohr and Mallouk [12] presented a more sophisticated theory by considering the pressure flow caused by the cross-channel pressure gradient in the screw channel and the effect of flight clearance on the drag flow. A detailed review of the early Newtonian theories was provided by Tadmor and Klein [1]. For extruder screws with “normal” clearances, the velocity profile in the flight clearance was shown to be determined mainly by the drag-flow component and influenced only marginally by the transverse pressure gradient.

Taking the shear-thinning flow behavior of polymer melts into account, Rauwendaal and Housz [32] used a numerical approach to readdress the flow characteristics in the flight clearance. For large flight clearances and small power-law indices, a significant influence of the transverse pressure flow was identified. Further, the total power consumption of the screw was shown to be affected considerably by the power consumption in the clearance. To account for the effect of leakage on throughput, corrections were made to (i) the transverse flow in the screw channel and (ii) the net flow rate. Focusing on pressure-generating metering zones, their analysis omitted overridden functional zones that are subject to a negative axial pressure gradient.

Including non-isothermal effects, Meyer et al. [33] numerically evaluated the temperature development in the flight clearance. Taking a pure drag flow into account, the results indicated that the thermal development length is generally smaller than the available gap length. When predicting the velocity and temperature distribution at the exit of the flight clearance, the flow can therefore be considered fully developed. To investigate the effect of leakage flow on the temperature distribution in the screw channel, Pittman and Rashid [34] and Rauwendaal [35] carried out numerical analyses of two- and three-dimensional power-law-model-based flows.

### 1.3. Research Approach

This work presents a new modeling approach to including leakage effects in the analysis of melt-conveying zones. Rather than directly correcting the drag and pressure flows, we developed new analytical regressions to predict the flow rate and dissipation locally in the leakage gap. In the construction of the leakage-flow models, particular attention was paid to their usability in our screw-simulation routine for predicting the conveying behavior of melt-conveying zones. For detailed information, the reader is referred to the following articles and PhD-theses: Aigner [36], Marschik et al. [37,38,39], Roland et al. [40], and Luger [41].

The main idea of the simulation approach is based on network theory: To reduce the complexity of the flow situation, the screw channel is subdivided into very small interconnected segments of constant cross-section and physical parameters. These sections are represented by network elements that consist of a source and a resistance connected in parallel. Using melt-conveying models from the literature, the network elements describe the flow locally in the screw channel. Analogously to electrical circuits, the network elements are connected via nodal points to form an equivalent flow network, whose flow rates and pressure differences are evaluated by means of nodal analysis. In contrast to time-consuming numerical simulations based on computational fluid dynamics, the method iteratively solves a linearized set of network equations and is therefore significantly faster.

**Figure 1:**
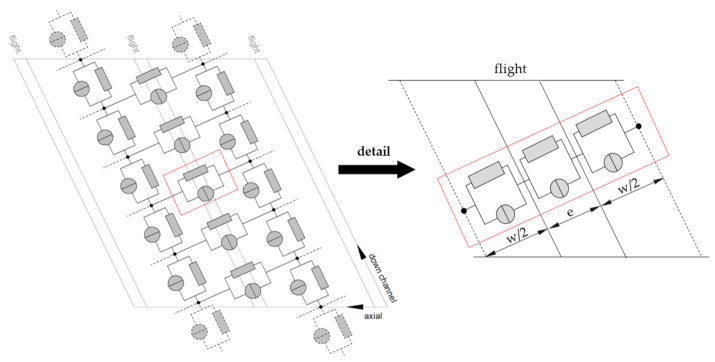
The development of leakage-flow models to predict flow rate and dissipation in the leakage gap of a discretized screw channel.

To illustrate the objective of this research, Figure 1 shows a flow network of a conventional metering zone, which consists of down- and cross-channel elements. Due to the changes in channel depth in the transverse direction, the latter is initialized with three network elements in series: (a) one from the channel center to the pushing flight, (b) one over the flight clearance, and (c) one from the trailing flight to the channel center one turn behind. The extrusion literature provides numerous theories for predicting the characteristics of the down-channel flow. Accurate models for the analysis of leakage effects, in contrast, remain elusive.

**Figure 2:**
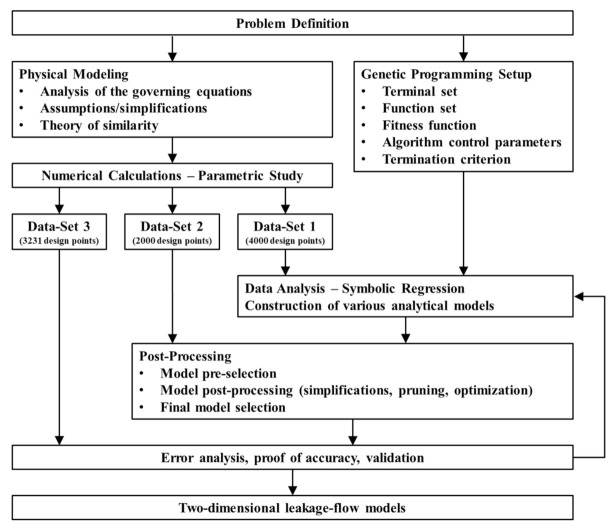
Schematic work-flow chart of the hybrid modeling approach, including analytical, numerical, and data-based modeling.

A flow chart of our work is given in Figure 2. First, we described mathematically the flow under consideration, converted the model into a dimensionless form, and carried out a detailed analysis of the governing equations. The dimensionless model was then solved numerically to evaluate flow rate and dissipation for a large number of physically independent setups. To remove the need for numerical methods, we derived approximate equations for flow rate and dissipation, using symbolic regression based on genetic programming. The structure of the regression models and their parameters were optimized iteratively until we obtained satisfying prediction accuracies. The new regressions were designed to be able to describe the properties of the network elements positioned in the flight gap.

## 2. Model Development

We used a hybrid modeling approach to derive approximate equations for the flow rate and the dissipation in the flight clearance. The method incorporates (i) analytical, (ii) numerical, and (iii) data-based modeling techniques. A detailed description of the main characteristics of our hybrid modeling approach was provided in [42].

### 2.1. Analytical Modeling

#### 2.1.1. Problem Definition

**Figure 3:**
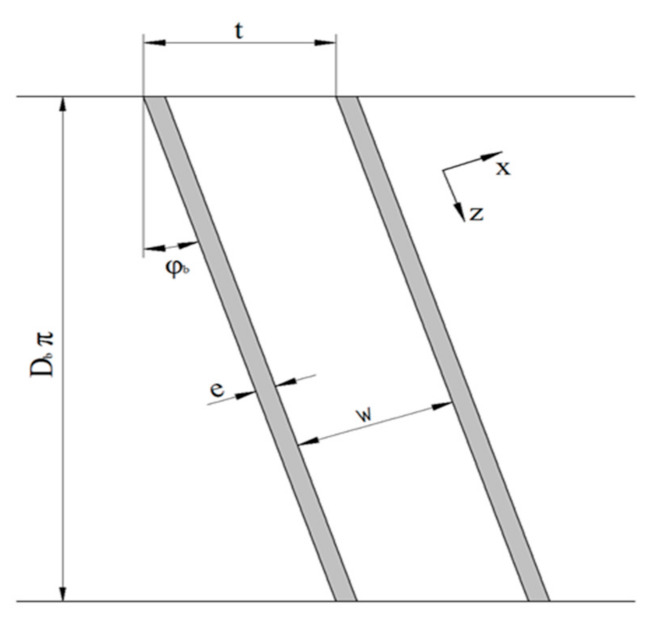
The top view of the unwound screw channel.

We applied the flat-channel approximation to simplify the helical reference frame of the screw. The screw channel is unwound and considered as a rectangular flow channel covered by an infinite flat plate. The top view of the unwound screw channel is shown in Figure 3. The simplified channel representation is based on a Cartesian coordinate system: x is the cross-channel direction and  z the down-channel direction. Avoiding cylindrical coordinates, the effect of channel curvature is ignored. The validity of the flat-plate model was discussed critically by Sun and Rauwendaal [43] and Roland et al. [44] for Newtonian and non-Newtonian polymer melts, respectively. Both studies confirmed the correctness of the approximation for small channel-depth-to-diameter ratios. This geometrical precondition is particularly fulfilled in the flight clearance. For conventional extruder screws with standard clearances, the ratio is typically in the range of $\delta /D_{b}=0.001$. As a result, the error introduced by unwinding the screw channel at the radial position of the barrel is small. The influence of channel curvature can hence be ignored. The following parameters were used to describe the geometry of the screw: $D_{b}$ is the screw diameter, t the screw pitch, e the flight width, w the channel width, and $\varphi_{b}$ the pitch angle.

Drawing on the traditional pumping model, we reversed the kinematics of the process; that is, the screw is stationary, and the barrel moves at circumferential speed $v_{b}$:

(1) $$v_{b}=D_{b}\;\pi \;N,$$

where N is the screw speed. The barrel velocity can be decomposed into a cross- and a down-channel component, $v_{b,x}$ and $v_{b,z}$:

(2) $$v_{b,x}=v_{b}\;sin\left(\varphi_{b}\right);\;\;\;\;\;\;\;\;\;\;\;\;\;\;\;\;\;\;\;\;v_{b,z}=v_{b}\;cos\left(\varphi_{b}\right);$$

(3) $$tan\left(\varphi_{b}\right)=\frac{t}{D_{b}\;\pi }.$$

**Figure 4:**
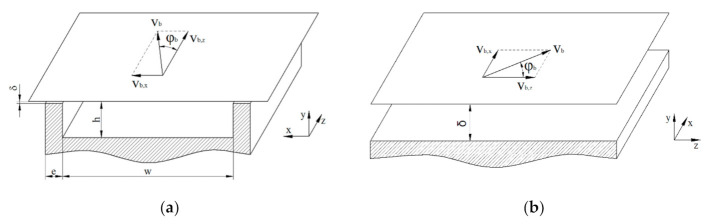
Flat-plate approximation of the unwound screw channel (**a**) and the flight clearance (**b**). In the representation of the flight clearance, the flow channel was rotated clockwise by 90°: x is the direction across and z the direction along the clearance.

Unlike previous studies that investigated the effect of the flight clearance [9,12,31,32], we locally analyzed the flow between flight land and barrel surface. Figure 4 illustrates the flat-plate model of the screw channel and the flight clearance.

The space through which the leakage flow occurs can be considered infinitely wide, resulting in a two-dimensional flow. Note that the coordinate system is the same in both cases. For convenience, however, the flow channel of the flight clearance was rotated clockwise by 90°: (i) x is the direction across the clearance, and (ii) z is the direction along the clearance. The channel gap between barrel surface and flight land is given by the clearance δ.

#### 2.1.2. Governing Equations

In the first step, we derived the governing equations to mathematically describe the flow through the flight clearance. To this end, we made the following assumptions: (i) the flow is independent of time, fully developed, and isothermal, (ii) the fluid is incompressible and wall-adhering, and (iii) gravitational forces are ignored. A detailed discussion on the validity of these commonly-applied assumptions was provided by Tadmor and Klein [1]. The velocity field was reduced to a two-dimensional flow:

(4) $$v=\left(\begin{array}{c} v_{x}\left(y\right)\\ 0\\ v_{z}\left(y\right) \end{array}\right).$$

Assuming a fully developed flow, the velocity components are functions of the channel-height coordinate y only; that is, the two-dimensional formulation is not capable of predicting any flow rate variations in the x- and z-directions. Meyer et al. [33] confirmed the validity of the assumption for both Newtonian and power-law fluids. Due to the low velocities and high viscosities of extruder flows, the Reynolds number is usually small (Re≪1), and the flow can be considered laminar [28,30]. Viscous forces being dominant over inertial forces, we reduced the problem to Stokes flow. By omitting time derivates, we ended up with the following simplified momentum equations:

(5) $$\frac{\partial p}{\partial x}=\frac{\partial \tau_{yx}}{\partial y}.$$

(6) $$\frac{\partial p}{\partial z}=\frac{\partial \tau_{yz}}{\partial y}.$$

In our isothermal theory, the momentum equations are uncoupled from the energy equation, which allowed us to evaluate viscous dissipation from the velocity field subject to the following boundary conditions:

(7) $$v_{x}\left(y=0\right)=0.\;\;\;\;\;\;\;\;\;\;\;\;\;\;\;\;\;\;\;\;v_{x}\left(y=\delta \right)=v_{b,x}.$$

(8) $$v_{z}\left(y=0\right)=0.\;\;\;\;\;\;\;\;\;\;\;\;\;\;\;\;\;\;\;\;v_{z}\left(y=\delta \right)=v_{b,z}.$$

The rheological behavior of the polymer melt was expressed by a nonlinear constitutive equation that relates the stress tensor τ to the rate-of-deformation tensor D, which is given by the symmetric part of the velocity-gradient tensor L:

(9) τ=2 η D.

(10) $$D=\frac{1}{2}\;\left(L+L^{T}\right).\;\;\;\;\;\;\;\;\;\;\;\;\;\;\;\;\;\;\;\;L=\nabla v.$$

For most polymer melts, the viscosity is strongly dependent on the velocity gradients in the flow field, rendering them shear thinning. To describe the shear-rate dependency of the viscosity, we applied a power-law model, where K is the consistency and n the power-law index. The magnitude of shear rate $\left|\dot{\gamma }\right|$ is related to the second invariant of the rate-of-deformation tensor:

(11) $$\eta =K{\left|\dot{\gamma }\right|}^{n-1}.$$

(12) $$\left|\dot{\gamma }\right|=\sqrt{2\left(D:D\right)}={\left[{\left(\frac{\partial v_{x}}{\partial y}\right)}^{2}+{\left(\frac{\partial v_{z}}{\partial y}\right)}^{2}\right]}^{\frac{1}{2}}.$$

With these definitions, the shear stresses were obtained from:

(13) $$\tau_{yx}=\eta \frac{\partial v_{x}}{\partial y}=K{\left[{\left(\frac{\partial v_{x}}{\partial y}\right)}^{2}+{\left(\frac{\partial v_{z}}{\partial y}\right)}^{2}\right]}^{\frac{n-1}{2}}\frac{\partial v_{x}}{\partial y}.$$

(14) $$\tau_{yz}=\eta \frac{\partial v_{z}}{\partial y}=K{\left[{\left(\frac{\partial v_{x}}{\partial y}\right)}^{2}+{\left(\frac{\partial v_{z}}{\partial y}\right)}^{2}\right]}^{\frac{n-1}{2}}\frac{\partial v_{z}}{\partial y}.$$

Finally, the momentum equations were rewritten as:

(15) $$\frac{\partial p}{\partial x}=\frac{\partial }{\partial y}\left\{K{\left[{\left(\frac{\partial v_{x}}{\partial y}\right)}^{2}+{\left(\frac{\partial v_{z}}{\partial y}\right)}^{2}\right]}^{\frac{n-1}{2}}\frac{\partial v_{x}}{\partial y}\right\}.$$

(16) $$\frac{\partial p}{\partial z}=\frac{\partial }{\partial y}\left\{K{\left[{\left(\frac{\partial v_{x}}{\partial y}\right)}^{2}+{\left(\frac{\partial v_{z}}{\partial y}\right)}^{2}\right]}^{\frac{n-1}{2}}\frac{\partial v_{z}}{\partial y}\right\}.$$

These coupled nonlinear partial differential equations are the governing equations of our model. In combination with the boundary conditions in Equations (7) and (8), the equation system describes the velocity field of the two-dimensional flow. Since, generally, valid closed-form analytical solutions are unknown, the shooting method was used to calculate the velocity field and the volume flow rate $\dot{V}$ per unit width and total viscous dissipation per unit area (${\dot{q}}_{diss}=\tau \;:L$):

(17) $$\dot{V}=\overset{\delta }{\underset{0}{\int }}v_{x}\left(y\right)dy.$$

(18) $${\dot{q}}_{diss}=\eta \left[{\left(\frac{\partial v_{x}}{\partial y}\right)}^{2}+{\left(\frac{\partial v_{z}}{\partial y}\right)}^{2}\right].$$

(19) $${\dot{Q}}_{diss}=\overset{\delta }{\underset{0}{\int }}{\dot{q}}_{diss}\left(y\right)dy.$$

#### 2.1.3. Theory of Similarity

Before the flow equations were solved numerically, the model was converted into a dimensionless form, using the theory of similarity, for the following reasons [26,27,28,29,30]: (i) Two systems described by the same dimensionless quantities are similar; (ii) varying any one of the independent input parameters changes the physical conditions of the flow; (iii) solutions obtained for a specific set of dimensionless input parameters apply to all dimensional variations that yield the same dimensionless input parameters.

We introduced the following dimensionless spatial coordinate and fluid velocities:

(20) $$\xi =\frac{y}{\delta }.\;\;\;\;\;\;\;\;\;\;\;\;\;\;\;\;\;\;\;\;v_{x}=\frac{v_{x}}{v_{b,x}}\;\;\;\;\;\;\;\;\;\;\;\;\;\;\;\;\;\;v_{z}=\frac{v_{z}}{v_{b,x}}.$$

These definitions were used to derive dimensionless parameters for shear rate and viscosity in the flight clearance:

(21) $$\eta_{\delta }^{*}=\frac{\eta \;\delta^{n-1}}{K\;v_{b,x}^{n-1}}={\left|{\dot{\gamma }}^{*}\right|}_{\delta }^{n-1}={\left[{\left(\frac{\partial v_{x}}{\partial \xi }\right)}^{2}+{\left(\frac{\partial v_{z}}{\partial \xi }\right)}^{2}\right]}^{\frac{n-1}{2}}.$$

Similarly, the momentum equations were transformed into dimensionless form:

(22) $$6\;\Pi_{p,x}^{\delta }=\frac{\partial }{\partial \xi }\left\{{\left[{\left(\frac{\partial v_{x}}{\partial \xi }\right)}^{2}+{\left(\frac{\partial v_{z}}{\partial \xi }\right)}^{2}\right]}^{\frac{n-1}{2}}\frac{\partial v_{x}}{\partial \xi }\right\};$$

(23) $$6\;\Pi_{p,z}^{\delta }=\frac{\partial }{\partial \xi }\left\{{\left[{\left(\frac{\partial v_{x}}{\partial \xi }\right)}^{2}+{\left(\frac{\partial v_{z}}{\partial \xi }\right)}^{2}\right]}^{\frac{n-1}{2}}\frac{\partial v_{z}}{\partial \xi }\right\},$$

where the parameters $\Pi_{p,x}^{\delta }$ and $\Pi_{p,z}^{\delta }$ are dimensionless pressure gradients:

(24) $$\Pi_{p,i}^{\delta }=\frac{\frac{\partial p}{\partial i}\;\delta^{n+1}}{6\;K\;v_{b,x}^{n}},\;\;\;\;\;\;\;\;\;\;i=x,z.$$

In addition, the boundary conditions were rewritten to:

(25) $$\;\;\;\;\;\;\;\;\;\;\;v_{x}\left(\xi =0\right)=0.\;\;\;\;\;\;\;\;\;\;\;\;\;\;\;\;\;\;\;\;v_{x}\left(\xi =1\right)=1.$$

(26) $$v_{z}\left(\xi =0\right)=0.\;\;\;\;\;\;\;\;\;\;\;\;\;\;\;\;\;\;\;\;v_{z}\left(\xi =1\right)=tan\left(90{}^{\circ}-\varphi_{b}\right).$$

Solving the dimensionless momentum Equations (22) and (23) in combination with the boundary conditions (25) and (26) required an additional mathematical constraint to be defined. Previous theories that modeled two-dimensional flows of polymer melts in infinitely wide screw channels considered the cross-channel net flow to be zero [22,26]. Physically, this means that leakage flow across the screw flights was ignored. Since this assumption is not reasonable in the analysis presented here, we instead assumed the pressure gradient in the z-direction to be zero. In our local formulation, this implies that the corresponding flow component is governed exclusively by the rotation of the screw:

(27) $$\frac{\partial p}{\partial z}=0\;\;\;\;\;\to \;\;\;\;\;\Pi_{p,z}^{\delta }=0.$$

Our assumption is based on the results of a similar study [26], in which the authors examined the flow of polymer melts in infinitely wide screw channels. For a variety of screw designs and processing conditions, the dimensionless down-channel pressure gradient along the screw channel $\Pi_{p,z}^{}$ was shown to be within the range of (−1.0;1.0). Rather than using the channel depth h, our theory requires the flight clearance δ to be predefined for the calculation of the dimensionless pressure gradient in the z-direction. Since the latter is significantly smaller, the dimensionless down-channel pressure gradient along the flight clearance $\Pi_{p,z}^{\delta }$ converges to zero.

A comparison of typical parameter values of $\Pi_{p,z}^{}$ and $\Pi_{p,z}^{\delta }$ is given in Table 1. The results indicate the different orders of magnitude of the parameters.

Finally, we derived dimensionless parameters for the volume flow rate $\Pi_{V}^{\delta }$ and the total viscous dissipation $\Pi_{Q}^{\delta }$:

(28) $$\Pi_{V}^{\delta }=\frac{2\;\dot{V}}{\delta \;v_{b,x}}=2\overset{1}{\underset{0}{\int }}v_{x}\left(\xi \right)\;d\xi .$$

(29) $$\Pi_{Q}^{\delta }=\frac{{\dot{Q}}_{diss}\;\delta^{n}}{K\;v_{b,x}^{n+1}}=\overset{1}{\underset{0}{\int }}{\left[{\left(\frac{\partial v_{x}}{\partial \xi }\right)}^{2}+{\left(\frac{\partial v_{z}}{\partial \xi }\right)}^{2}\right]}^{\frac{n+1}{2}}\;d\xi .$$

Our dimensionless model has three independent input parameters that completely describe the physics of the flow: (i) the screw-pitch ratio $t/D_{b}$, indicated by $tan\left(\varphi_{b}\right)$, (ii) the power-law index n, and (iii) a dimensionless pressure gradient across the flight clearance $\Pi_{p,x}^{\delta }$. The first is part of the boundary conditions (25), while the second and third are included in the momentum Equations (22) and (23).

#### 2.1.4. Set-Up of Parametric Study

We created four sets of physically independent design points by varying the input parameters $t/D_{b}$, n, and $\Pi_{p,x}^{\delta }$ (Table 2, Table 3 and Table 4). For all sets, the screw-pitch ratio and the power-law index were varied within the ranges (0.5–2.47) and (0.2–1.0), respectively. These ranges include almost all extruder screws and polymer melts in industrial use. The variation of the dimensionless pressure gradient across the flight clearance $\Pi_{p,x}^{\delta }$ was adjusted case by case. To determine the industrially relevant parameter ranges, we carried out screw calculations for various types of extruder screws, using our network-based routine [1,2,3,4,5,6,7,8,9,10,11,12,13,14,15,16,17,18,19,20,21,22,23,24,25,26,27,28,29,30,31,32,33,34,35,36,37,38,39,40].

For conventional extruder screws with normal clearances ($\delta \approx 0.001\;D_{b}$), the dimensionless pressure gradient is relatively small. Its magnitude, however, increases if the extruder screw is constructed with an undercut flight. This situation can be found, for example, in barrier screws. High dimensionless pressure gradients arise in the context of high-performance screws, such as energy-transfer screws, in which screw flights are, by design, undercut to promote cross-channel mixing. In addition, the direction of leakage flow depends on the conveying characteristics of the screw. For pressure-generating functional zones, leakage flow typically reduces the throughput. For strongly overridden functional zones, in contrast, polymer melt passing through the clearance is forced towards the screw tip to increase the net flow rate of the processing machine. This effect can be increased if the flight clearance is undercut.

For Data Set 1, the dimensionless pressure gradient was varied within the range (−1.0;1.0). Since pressure development also depends on the shear-thinning nature of the polymer melt, the minima, maxima, and increments for Data Sets 2–4 were based on the power-law index. In total, we created 11,781 design points. Finally, Data Sets 1–4 were merged, and design points with multiple occurrences were deleted, which yielded 9231 physically independent setups.

### 2.2. Numerical Modeling

#### 2.2.1. Numerical Solution Procedure

In the next step, we numerically evaluated the target variables (dimensionless volume flow rate $\Pi_{V}^{\delta }$ and dimensionless dissipation $\Pi_{Q}^{\delta }$) of our model for all 9,231 physically independent setups, using the shooting method. To this end, we calculated the velocity field in the flight clearance by solving the governing equations of our dimensionless model. A detailed description of the numerical solution procedure was given in [26]. Transforming the boundary value into an initial-value problem, we derived explicit forms of the dimensionless momentum equations:

(30) $$\frac{\partial v_{x}}{\partial \xi }=\frac{1}{\eta_{\delta }^{*}}\left(6\;\Pi_{p,x}^{\delta }\xi +C_{1}\right);$$

(31) $$\frac{\partial v_{z}}{\partial \xi }=\frac{C_{2}}{\eta_{\delta }^{*}},$$

where the dimensionless viscosity was rewritten as:

(32) $$\eta_{\delta }^{*}={\left[{\left(6\;\Pi_{p,x}^{\delta }\xi +C_{1}\right)}^{2}+C_{2}^{2}\right]}^{\frac{n-1}{2n}}.$$

The initial estimates of the integration constants $C_{1}$ and $C_{2}$ were taken from the Newtonian solution. Applying the Simpsons rule yielded the following equations for the velocity profiles:

(33) $$v_{x}\left(\xi \right)=\underset{0}{\underbrace{v_{x}\left(\xi =0\right)}}+\overset{1}{\underset{0}{\int }}\frac{\partial v_{x}}{\partial \xi }d\xi .$$

(34) $$v_{z}\left(\xi \right)=\underset{0}{\underbrace{v_{z}\left(\xi =0\right)}}+\overset{1}{\underset{0}{\int }}\frac{\partial v_{z}}{\partial \xi }d\xi .$$

To iteratively solve the unknowns, we used a Newton-Raphson scheme:

(35) $$x_{n+1}=x_{n}-J{\left(x_{n}\right)}^{-1}\left[f\left(x_{n}\right)-f\left(x\right)\right],$$

where x is the vector of unknowns, J the Jacobian matrix, and f is the vector of boundary conditions:

(36) $$\left(\begin{array}{c} C_{1,n+1}\\ C_{2,n+1} \end{array}\right)=\left(\begin{array}{c} C_{1,n}\\ C_{2,n} \end{array}\right)-{\left(\begin{array}{cc} \frac{\partial v_{x,1}}{\partial C_{1}} & \frac{\partial v_{x,1}}{\partial C_{2}}\\ \frac{\partial v_{z,1}}{\partial C_{1}} & \frac{\partial v_{z,1}}{\partial C_{2}} \end{array}\right)}^{-1}\left[\left(\begin{array}{c} v_{x,1,n}\\ v_{z,1,n} \end{array}\right)-\left(\begin{array}{c} tan\left(\varphi_{b}\right)\\ 1 \end{array}\right)\right].$$

The velocity boundary conditions at the barrel surface will not be met unless the initial values are perfect. The converged solutions for the velocity profiles were then used to determine the dimensionless target variables $\Pi_{V}^{\delta }$ and $\Pi_{Q}^{\delta }$.

For all calculations, the dimensionless channel height was divided into 1000 equidistant segments. A solution was considered converged if the difference in dimensionless volume flow rate $\Pi_{V}^{\delta }$ between two iterations was smaller than 10^-8^. Previous analyses have shown that these settings are sufficient to obtain mesh-independent results for our target variables [26].

#### 2.2.2. Numerical Results

Our parametric design study encompassing 9,231 independent setups provided numerical solutions for the dimensionless volume flow rate $\Pi_{V}^{\delta }$ and the dissipation $\Pi_{Q}^{\delta }$ in the flight clearance as functions of the dimensionless input parameters $t/D_{b}$, n, and $\Pi_{p,x}^{\delta }$.

**Figure 5:**
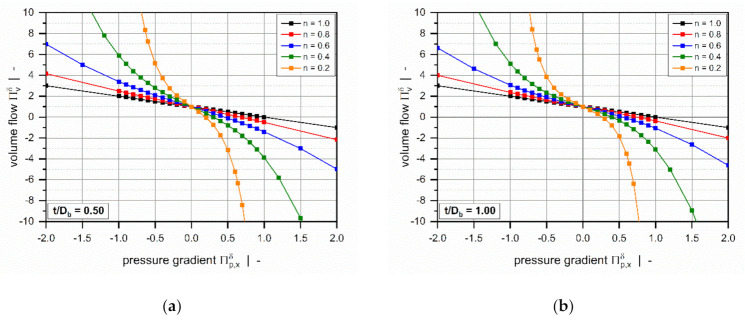
The volume flow rate $\Pi_{V}^{\delta }$ as a function of pressure gradient $\Pi_{p,x}^{\delta }$: Influence of power-law index for $t/D_{b}=0.5$ (**a**) and $t/D_{b}=1.0$ (**b**).

Figure 5 shows the dimensionless volume flow rate as a function of the dimensionless pressure gradient across the flight clearance for various power-law indices. For all setups, the curves are symmetrical about the point of pure drag flow ($\Pi_{p,x}^{\delta }=0$); that is, an equidistant increase in the pressure gradient (positive or negative) affects the magnitude of the dimensionless volume flow rate equally.

The power-law index is a measure of the shear-thinning behavior of the polymer melt: the lower the power-law index, the more shear-thinning is the fluid. Assuming a Newtonian fluid, the widely-known linear behavior is evident: For $\Pi_{p,x}^{\delta }=0$ (pure drag flow), the curve satisfies $\Pi_{V}^{\delta }=1$, while for $\Pi_{p,x}^{\delta }=1$, the zero-throughput condition is fulfilled $\Pi_{V}^{\delta }=0$.

Generally, the volume flow rates become negative if a critical pressure gradient is reached. From a mathematical viewpoint, this means that the direction of flow changes to the negative x-direction. Physically, it implies that the pressure flow caused by the pressure build-up across the clearance exceeds the drag flow, which yields a negative net throughput. According to our model definition, this behavior is subject to strongly overridden melt-conveying zones. Positive flow rates, in contrast, are found in pressure-generating metering zones, in which the leakage flow reduces the net throughput. With decreasing power-law index, the critical pressure gradient shifts to lower values.

The curves become increasingly nonlinear and pressure-sensitive with decreasing power-law index. Two effects are evident: (i) For a given dimensionless volume flow rate, the more the dimensionless pressure gradient (positive and negative) increases, the less shear-thinning the fluid. (ii) For highly shear-thinning polymer melts, small variations in the pressure gradient can lead to pronounced variations in the volume flow rate.

**Figure 6:**
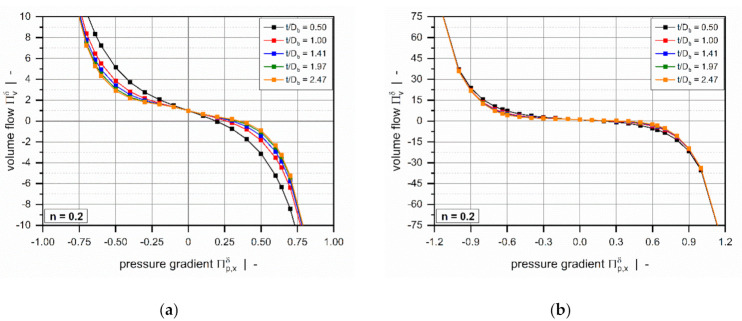
The volume flow rate $\Pi_{V}^{\delta }$ as a function of pressure gradient $\Pi_{p,x}^{\delta }$: Influence of screw-pitch ratio for n=0.2. The scaling of the diagrams was adjusted to better visualize the influence for smaller pressure gradients (**a**) and larger pressure gradients (**b**).

The influence of the screw-pitch ratio on the dimensionless volume flow rate is less pronounced (Figure 6). For positive pressure gradients, the target variable increases with increasing screw-pitch ratio, while the opposite behavior is evident for negative pressure gradients. This effect is caused by the influence of the flow along the flight clearance (in the z-direction) on the deformation rates, which becomes more pronounced the lower the screw-pitch ratio.

For markedly positive or negative dimensionless pressure gradients (Figure 6b), the effect of the screw-pitch ratio decreases significantly since the flow is governed mainly by the pressure gradient across the flight clearance.

**Figure 7:**
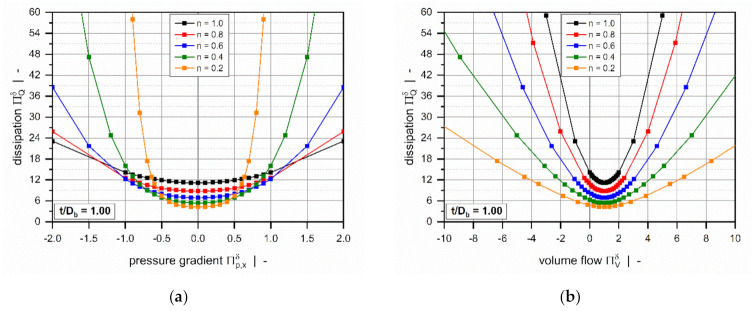
Dimensionless dissipation $\Pi_{Q}^{\delta }$ as a function of the dimensionless pressure gradient $\Pi_{p,x}^{\delta }$ (**a**) and as a function of the dimensionless volume flow rate $\Pi_{V}^{\delta }$. (**b**) The influence of the power-law index for a square-pitched screw with $t/D_{b}=1.0$.

Figure 7 illustrates the influence of the power-law index on the dimensionless dissipation for a square-pitched screw with $t/D_{b}=1$. Viscous dissipation is mainly responsible for the temperature development in the channel. Due to inner friction, mechanical energy is converted into heat, causing a rise in melt temperature.

The dimensionless dissipation can be plotted as a function of either the dimensionless pressure gradient (Figure 7a) or the dimensionless volume flow rate (Figure 7b). For both representations, the curves are again symmetrical about the point of pure drag flow ($\Pi_{p,x}^{\delta }=0$ or $\Pi_{V}^{\delta }=1$), where the target variable reaches a minimum. In general, the dimensionless dissipation increases if the pressure flow contributes to the flow characteristics; that is, the higher the dimensionless pressure gradient, the more pronounced is the frictional heat generation. Similarly, dissipation becomes highly dependent on the power-law index for strongly pressure-generating or pressure-consuming flows.

Three effects are observed: (i) For moderate pressure gradients, the more dimensionless dissipation decreases, the more shear-thinning the polymer melt. (ii) For higher magnitudes, in contrast, the more frictional heat generation decreases, the more Newtonian the fluid. (iii) For constant dimensionless volume flow rates, viscous heating increases with the increasing power-law index.

**Figure 8:**
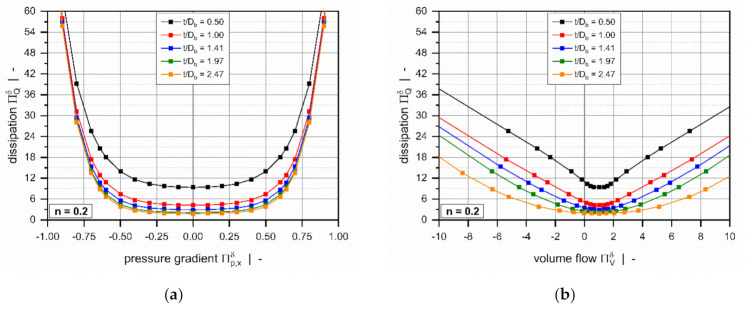
Dimensionless dissipation $\Pi_{Q}^{\delta }$ as a function of the dimensionless pressure gradient $\Pi_{p,x}^{\delta }$ (**a**) and as a function of the dimensionless volume flow rate $\Pi_{V}^{\delta }$. (**b**) The influence of the screw-pitch ratio for a polymer melt with power-law index n=0.2.

Figure 8 shows the influence of the screw-pitch ratio on the dimensionless dissipa-tion for a polymer melt with power-law index n = 0.2. For both constant dimensionless pressure gradients (Figure 8a) and constant dimensionless flow rates (Figure 8b), viscous dissipation increases with decreasing screw-pitch ratio. This result is again caused by the effect of transverse flow in the leakage gap. The influence of the screw-pitch ratio on dimensionless dissipation vanishes almost completely in strongly pressure-generating and pressure-consuming flows.

**Figure 9:**
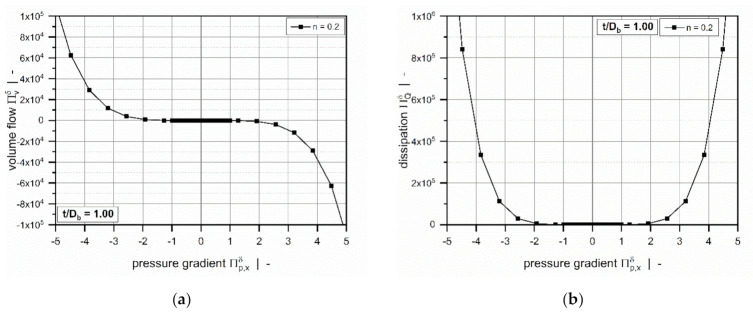
The dimensionless volume flow rate $\Pi_{V}^{\delta }$ (**a**) and dimensionless dissipation $\Pi_{Q}^{\delta }$ (**b**) as functions of the dimensionless pressure gradient $\Pi_{p,x}^{\delta }$ for a square-pitched screw with $t/D_{b}=1.0$ and a polymer melt with the power-law index n=0.2.

To represent the diverse characteristics of the leakage flow, we considered a wide range of dimensionless pressure gradients. Especially for highly shear-thinning polymer melts with low power-law indices, our extended dataset caused a significant nonlinear increase in the target variables, yielding values higher than $\left|\Pi_{V}^{\delta }\right|>10^{5}$ and $\left|\Pi_{Q}^{\delta }\right|>10^{6}$, as illustrated in Figure 9. Recently, we have shown that the parameters are limited to $\left|\Pi_{V}^{}\right|<40$ and $\left|\Pi_{Q}^{}\right|<140$ when analyzing the flow in metering channels [26]. This comparison illustrates the increased complexity of the following symbolic regression analysis.

### 2.3. Data-Based Modeling

#### 2.3.1. Symbolic Regression Analysis

Removing the need for numerical simulations required two analytical regressions that accurately predict the numerical solutions of our parametric design study. For this reason, we approximated the numerical solutions of our parametric design study analytically by using symbolic regression based on genetic programming implemented in the open-source software HeuristicLab (Hagenberg, Austria) [45]. This data-based modeling approach searches the space of mathematical expressions to find regressions that relate sets of input and output data. For detailed information, the reader is referred to [46,47].

To reduce the ranges of our target variables and the nonlinearities in our dataset, we took advantage of the following phenomenon: For large dimensionless pressure gradients, our numerical results showed that the influence of the screw-pitch ratio converges to zero, causing the pressure flow across the flight clearance to dominate the overall flow characteristics. Ignoring the effect of the flow in the *z*-direction, we used the following analytical relationships to approximate the dimensionless volume flow rate and the dissipation [26]:

(37) $$\Pi_{V,app}^{\delta }=1-sign\left(\Pi_{p,x}^{\delta }\right)\frac{3^{\frac{1}{n}}\;n}{2\;n+1}{\left|\Pi_{p,x}^{\delta }\right|}^{\frac{1}{n}}.$$

(38) $$\Pi_{Q,app}^{\delta }=\frac{{\left(2n+1\right)}^{n}}{n^{n}}{\left|\Pi_{V}^{\delta }-1\right|}^{\left(1+n\right)}.$$

Considering the flow of a power-law fluid, the first relationship describes the dimensionless volume flow rate as a function of the pressure gradient by a linear superposition of a one-dimensional drag and pressure flow. The second relationship, in contrast, approximates the dimensionless dissipation as a function of the volume flow rate by a one-dimensional pressure flow. These parameters were used to create new target variables by correcting the numerical results:

(39) $$\Delta \Pi_{V}^{\delta }=\Pi_{V}^{\delta }-\Pi_{V,app}^{\delta }.$$

(40) $$\Delta \Pi_{Q}^{\delta }=\frac{\Pi_{Q}^{\delta }}{\Pi_{Q,app}^{\delta }+1}.$$

**Figure 10:**
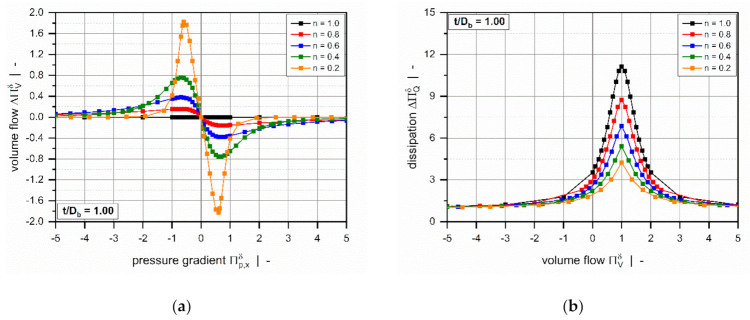
The corrected dimensionless volume flow rate $\Delta \Pi_{V}^{\delta }$ (**a**) and corrected dimensionless dissipation $\Delta \Pi_{Q}^{\delta }$ (**b**) as functions of the dimensionless volume flow rate $\Pi_{V}^{\delta }$ for a square-pitched screw with $t/D_{b}=1.0$ and a polymer melt with power-law index n=0.2.

Figure 10 illustrates the characteristics of the modified target variables for a square-pitched screw and various power-law indices. Rather than showing a strong nonlinear increase for increased dimensionless pressure gradients, both parameters reach a plateau, thereby decreasing the value range of the target variables. Again, the curves are symmetrical about the point of pure drag flow ($\Pi_{p,x}^{\delta }=0$ or $\Pi_{V}^{\delta }=1$).

In the construction of the regression models, we randomly divided our dataset into three subsets: (i) a training set, (ii) a test set, and (iii) a validation set, including 4000, 2000, and 3231 design points, respectively. The first two subsets were employed to develop and optimize the symbolic regression solutions, while the third subset was used to validate the models against unseen numerical results not used in model development.

We applied the NSGA-II algorithm [48] to construct two regressions of the form:

(41) $$\Delta \Pi_{V}^{\delta }=f\left(\Pi_{p,x}^{\delta },\;n,\;\;t/D_{b}\right);$$

(42) $$\Delta \Pi_{Q}^{\delta }=f\left(\Pi_{V}^{\delta },\;n,\;\;t/D_{b}\right).$$

This multi-objective non-dominant genetic algorithm simultaneously optimizes model quality and complexity. The latter was adjusted by restricting (a) the model size to a maximum tree length of 100, and (b) the function set, which defines the functions used to generate symbolic regression solutions, to (i) constant, (ii) state variable, (iii) addition, (iv) multiplication and division, and (v) cosine and sine, thus limiting the search space of the regression analysis. Model optimization was driven by a constant optimization evaluator, which calculates Pearson $R^{2}$ of a symbolic regression solution and optimizes the constants used:

(43) $$R^{2}=1-\frac{\sum_{i=1}^{n}{\left(y_{i}-{\hat{y}}_{i}\right)}^{2}}{\sum_{i=1}^{n}{\left(y_{i}-\bar{y}\right)}^{2}},$$

where $y_{i}$ and ${\hat{y}}_{i}$ are the numerical and approximated results, respectively, and $\bar{y}$ is the mean of the numerical solutions.

For each model, we first performed 20 runs to generate a set of symbolic regression solutions, using the training and test data and then selected the most accurate approximation. To evaluate model quality, we carried out an error analysis for all subsets.

#### 2.3.2. Symbolic Regression Results

Our hybrid modeling approach provided two analytical regression for the corrected dimensionless volume flow rate $\Delta \Pi_{V}^{\delta }$ and the corrected dimensionless dissipation $\Delta \Pi_{Q}^{\delta }$:

(44) $$\Delta \Pi_{V}^{\delta }\left(\Pi_{p,x}^{\delta },\;n,\;t/D_{b}\right)=a_{00}+\frac{A_{1}+A_{2}\;A_{3}}{A_{4}+A_{5}+A_{6}};$$

(45) $$\Delta \Pi_{Q}^{\delta }\left(\Pi_{V}^{\delta },\;n,\;t/D_{b}\right)=b_{00}+\frac{B_{1}+\frac{B_{2}}{B_{3}}+B_{4}}{B_{5}+B_{6}},$$

where $A_{1}–A_{6}$ and $B_{1}–B_{6}$ are the subfunctions, which contain 25 ($a_{00}–a_{24}$) and 31 ($b_{00}–b_{30}$) coefficients, respectively. The subfunctions and their coefficients are given in the Appendix A. Note that the regressions predict the corrected target variables, while the actual parameters result from:

(46) $$\Pi_{V}^{\delta }=\Delta \Pi_{V}^{\delta }+\Pi_{V,app}^{\delta }.\;\;\;\;\;\;\;\;\;\;\;\;\;\;\;\;\;\;\;\;\Pi_{Q}^{\delta }=\Delta \Pi_{Q}^{\delta }\;\left(\Pi_{Q,app}^{\delta }+1\right).$$

Avoiding complex nested analytical functions, the models include only basic arithmetic operations and analytical functions. Due to their relatively simple mathematical structure, the approximations allow fast computation of the target variables without the need for further numerical simulations.

To evaluate the accuracy of the regression solutions, we carried out an error analysis for all subsets, including 9,231 design points in total. For this reason, we determined the mean absolute error ($AE_{mean}$), the maximum absolute error ($AE_{max}$), and the coefficient of determination ($R^{2}$). The accuracy of the dissipation model was additionally investigated by comparing the mean relative error ($RE_{mean}$) and the maximum relative error ($RE_{max}$):

(47) $$AE_{mean}=\frac{1}{N}\overset{n}{\underset{i=1}{\sum }}\left|y_{i}-{\hat{y}}_{i}\right|.\;\;\;\;\;\;\;\;\;\;\;\;\;\;\;\;\;\;\;AE_{max}=\max\;\left(\left|y_{i}-{\hat{y}}_{i}\right|\right).$$

(48) $$RE_{mean}=\frac{1}{N}\overset{n}{\underset{i=1}{\sum }}\frac{\left|y_{i}-{\hat{y}}_{i}\right|}{y_{i}}.\;\;\;\;\;\;\;\;\;\;\;\;\;\;\;\;\;\;\;\;RE_{max}=\max\;\left(\frac{\left|y_{i}-{\hat{y}}_{i}\right|}{y_{i}}\right).$$

**Figure 11:**
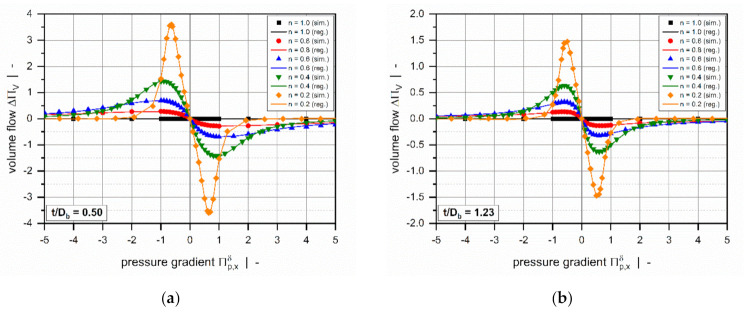
A comparison of approximated results obtained from $\Delta \Pi_{V}^{\delta }=f\left(t/D_{b},\;n,\;\Pi_{p,x}^{\delta }\right)\;$ and numerical solutions for $t/D_{b}=0.50$ (**a**) and $t/D_{b}=1.23$ (**b**). The points indicate numerical results, and the continuous lines approximated solutions.

**Figure 12:**
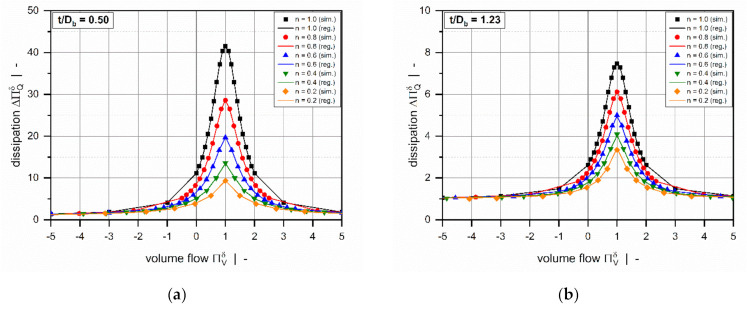
A comparison of approximated results obtained from $\Delta \Pi_{Q}^{\delta }=f\left(t/D_{b},\;n,\;\Pi_{V}^{\delta }\right)\;$ and numerical solutions for $t/D_{b}=0.50$ (**a**) and $t/D_{b}=1.23$ (**b**). The points indicate numerical results, and the continuous lines approximated solutions.

**Figure 13:**
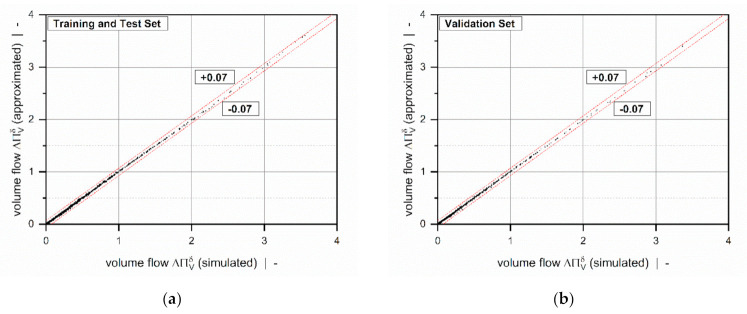
A scatter plot of $\Delta \Pi_{V}^{\delta }=f\left(t/D_{b},\;n,\;\Pi_{p,x}^{\delta }\right)$: training and test set (**a**) and validation set (**b**). The dashed lines indicate an absolute error of 0.07.

**Figure 14:**
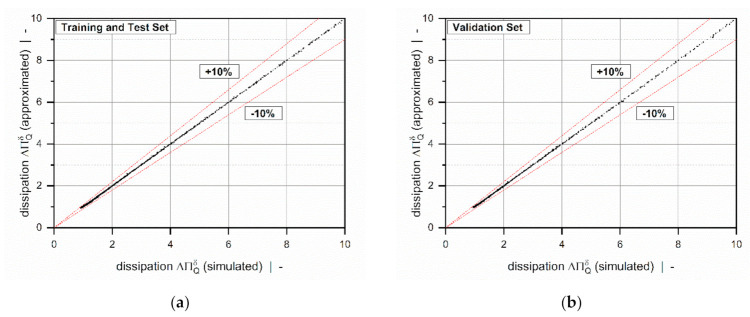
A scatter plot of $\Delta \Pi_{Q}^{\delta }=f\left(t/D_{b},\;n,\;\Pi_{V}^{\delta }\right)$: training and test set (**a**) and validation set (**b**). The dashed lines indicate a relative error of 10%.

Overviews of the results of the error measures are given in Table 5 and Table 6. Both models achieved a coefficient of determination $R^{2}>0.999$ for all subsets, which indicates excellent accuracy of the approximations. Since the results of the error measures of all subsets fall within a similar range, we conclude that overfitting was avoided. The low mean absolute and mean relative errors confirm the validity of the new models over the entire range of input parameters. Note that the design points of the validation set were not used for model development and therefore enabled an unbiased estimation of model quality. To demonstrate the performance of the regression models on the original numerical data, including 9231 design points, Table 7 shows the error measures of the final models in Equation (46). As the corrected target variables were constructed by using addition and multiplication, the models for the flow rate and dissipation exhibit the same absolute and relative errors, respectively. Comparisons of numerical and approximated results are illustrated in Figure 11 and Figure 12 for various setups. The points indicate numerical results, and the continuous lines approximated solutions. Further, Figure 13 and Figure 14 show a normalized representation of all design points in the form of scatter plots that compare numerical and approximated results for all subsets. Removing the need for numerical simulations, the new leakage-flow models can be used to include the effect of the flight clearance in the analysis of melt-conveying zones.

## 3. Conclusions

We have proposed novel analytical regression models for predicting the volume flow rate and the viscous dissipation rate in the flight clearance of single-screw extruders. Unlike theories that correct the drag and pressure flows in the traditional pumping model, these approximations were designed to locally evaluate the characteristics of the leakage flow. Using a local reference system of the flight clearance allowed us to relax a variety of modeling assumptions and, therefore, to increase the accuracy of the models.

The approach presented here combines analytical, numerical, and data-based modeling techniques. The governing flow equations were rewritten in a dimensionless form by applying the theory of similarity. Three physically independent dimensionless input parameters of the flow were identified: (i) the screw-pitch ratio $t/D_{b}$, (ii) the power-law index n, and (iii) a dimensionless pressure gradient across the flight clearance $\Pi_{p,x}^{\delta }$. These dimensionless parameters were varied to create 9,231 physically independent setups, whose volume flow rates and dissipations were evaluated numerically with the shooting method. The numerical solutions of the design study were then approximated by means of symbolic regression based on genetic programming. The hybrid modeling approach yielded two analytical relationships for volume flow rate and dissipation, the accuracy of which we have demonstrated in an error analysis, yielding a Pearson $R^{2}>0.999$ for both models. The first model showed a maximum absolute error of $AE_{max}=0.06735$, while the second model produces a maximum relative error of $RE_{max}=6.64\%$.

A novel feature of our theory is the flat-plate representation of the flight clearance. This local reference system gives rise to a two-dimensional flow in which the drag- and pressure flows in the cross- and down-channel directions are coupled due to the shear-rate-dependent viscosity of the power-law fluid. To be able to solve the governing flow equations, we introduced a mathematical constraint by omitting the pressure gradient along the flight clearance (in the z-direction). From a physical viewpoint, this means that the corresponding flow component is governed exclusively by the rotation of the screw. This assumption was shown to be valid if the flight clearance is significantly smaller than the channel depth (δ/h≪1).

To extend the applicability of the new leakage-flow models to a variety of screw designs and processing conditions in industrial use, our approach considered a significant range of dimensionless pressure gradients. Preliminary analyses indicated that these parameter ranges cover several orders of magnitude, depending on the depth of the leakage gap. Low values were observed for conventional screw designs with standard clearances ($\delta \approx 0.001\;D_{b}$), while high values were detected for high-performance screws with pronounced undercut distances between main and secondary flight (e.g., wave-dispersion screws). In addition, the calculations illustrated that the direction of leakage flow is governed mainly by the conveying characteristics of the extruder screw. For pressure-generating melt-conveying zones, leakage flow reduces the net flow of the processing machine. For strongly overridden functional zones, in contrast, leakage flow increases the net flow.

Since the diverse characteristics of the leakage flow were considered in the construction of the design points, the numerical results of our parametric study extend over several orders of magnitude. To decrease the parameter ranges for the symbolic regression analyses, two modified target variables were constructed by assuming that, for large dimensionless pressure gradients, the flow can be approximated mathematically by linear superposition of one-dimensional drag and pressure flows. The corrected parameters were designed to reach a plateau for increased dimensionless pressure gradients, thereby decreasing the nonlinearities in the dataset.

The novel leakage-flow models will be implemented in our screw-simulation routine based on network theory (presented in [1,2,3,4,5,6,7,8,9,10,11,12,13,14,15,16,17,18,19,20,21,22,23,24,25,26,27,28,29,30,31,32,33,34,35,36,37,38,39,40]). The aim is to accurately describe the properties of the network elements positioned in the leakage gap to include the effect of the flight clearance in the prediction of pumping capability and power consumption. Using the new leakage-flow models in the network calculation, the modeling approach is no longer limited to any specific geometrical design and allows fast and accurate analysis of various types of extruder screws, including both conventional and high-performance screws.

## Figures and Tables

**Table 1:** Dimensionless pressure gradients in the screw channel $\Pi_{p,z}^{}$ and in the flight clearance $\Pi_{p,z}^{\delta }$.

Parameter	Unit	Screw Channel	Flight Clearance
K	Pas^n^	1000	1000
n	-	0.3	0.3
D	mm	45	45
N	rpm	100	100
h or δ	mm	3.5	0.075
∂p/∂z	Pa/m	50·10^5^	50·10^5^
$\Pi_{p,z}^{\delta }$ or $\Pi_{p,z}^{\delta }$	-	0.82	0.006

**Table 2:** The ranges of variation for $t/D_{b}$, n, and $\Pi_{p,x}^{\delta }$ for Data Set 1.

Variable	Min	Max	Delta
$t/D_{b}$	0.50	2.47	variable
n	0.2	1.0	0.1
$\Pi_{p,x}^{\delta }$	-1.0	1.0	0.1

**Table 3:** The ranges of variation for $t/D_{b}$ and n for Data Sets 2 to 4.

Variable	Min	Max	Delta
$t/D_{b}$	0.50	2.47	variable
n	0.2	1.0	0.1

**Table 4:** The ranges of variation for $\Pi_{p,x}^{\delta }$ for Data Sets 2 to 4.

n	Data Set 2			Data Set 3			Data Set 4		
	Min	Max	Delta	Min	Max	Delta	Min	Max	Delta
1.0	−2000	2000	200	−40	40	4	−20	20	2
0.9	−2000	2000	200	−20	20	2	−10	10	1
0.8	−1000	1000	100	−20	20	2	−10	10	1
0.7	−400	400	40	−20	20	2	−10	10	1
0.6	−200	200	20	−10	10	1	−5	5	0.5
0.5	−80	80	8	−10	10	1	−5	5	0.5
0.4	−32	32	3.2	−5	5	0.5	−3	3	0.3
0.3	−12.8	12.8	1.28	−3	3	0.3	-	-	-
0.2	−6.4	6.4	0.64	-	-	-	-	-	-

**Table 5:** The error measures of $\Delta \Pi_{V}^{\delta }=f\left(t/D_{b},\;n,\;\Pi_{p,x}^{\delta }\right)$.

Quality Measure	Unit	Training Set	Test Set	Validation Set
$R^{2}$	-	0.99985	0.99983	0.99986
$AE_{mean}$	-	0.00536	0.00543	0.00545
$AE_{max}$	-	0.04511	0.06068	0.06735

**Table 6:** The error measures of $\Delta \Pi_{Q}^{\delta }=f\left(t/D_{b},\;n,\;\Pi_{V}^{\delta }\right)$.

Quality Measure	Unit	Training Set	Test Set	Validation Set
$R^{2}$	-	0.99999	0.99999	0.99999
$AE_{mean}$	-	0.00638	0.00698	0.00634
$AE_{max}$	-	00.15041	0.17306	0.20197
$RE_{mean}$	%	0.31	0.33	0.3
$RE_{max}$	%	6.65	6.29	6.64

**Table 7:** The error measures of $\Pi_{V}^{\delta }=f\left(t/D_{b},\;n,\;\Pi_{p,x}^{\delta }\right)$ and $\Pi_{Q}^{\delta }=f\left(t/D_{b},\;n,\;\Pi_{p,x}^{\delta }\right)$.

Quality Measure	Unit	$\Pi_{V}^{\delta }$	$\Pi_{Q}^{\delta }$
$R^{2}$	-	0.99985	0.99999
$AE_{mean}$	-	0.00513	1047.77
$AE_{max}$	-	0.06735	30270.9
$RE_{mean}$	%	-	0.31
$RE_{max}$	%	-	6.64

## Data Availability

The data presented in this study are available on request from the corresponding author.

## Appendix A

### Appendix A.1. Subfunctions of Equations (44) and (45)

(A1) $${\left(\frac{t}{D_{b}}\right)}_{eff}=\pi \;tan\left(90-arctan\left(\frac{t}{D_{b}\pi }\right)\right)\;$$

(A2) $$A_{1}=a_{01}\;\Pi_{p,x}^{\delta }{\left(\frac{t}{D_{b}}\right)}_{eff}\;$$

(A3) $$A_{2}=a_{02}\;\left(a_{03}+n\right)\;\left(a_{04}+n\right)\;\Pi_{p,x}^{\delta }\;\left(a_{05}+\left(a_{06}+n\right)\;\Pi_{p,x}^{\delta }{}^{2}+\left(a_{07}+a_{08}\;n\right){\left(\frac{t}{D_{b}}\right)}_{eff}\right).$$

(A4) $$A_{3}=n+\left(a_{09}+a_{10}\;\Pi_{p,x}^{\delta }{}^{2}\right){\left(\frac{t}{D_{b}}\right)}_{eff}.$$

(A5) $$A_{4}=a_{11}+a_{12}\;\Pi_{p,x}^{\delta }{}^{2}.$$

(A6) $$A_{5}=a_{13}\left(a_{14}+\Pi_{p,x}^{\delta }{}^{2}+a_{15}{\left(\frac{t}{D_{b}}\right)}_{eff}\right)\left(a_{16}\;\Pi_{p,x}^{\delta }{}^{2}+\Pi_{p,x}^{\delta }{}^{4}+a_{17}{\left(\frac{t}{D_{b}}\right)}_{eff}\right).$$

(A7) $$A_{6}=a_{18}\;n^{3}\;\left(a_{19}+n+a_{20}{\left(\frac{t}{D_{b}}\right)}_{eff}\right)+a_{21}\;n^{2}\;\left(a_{22}+n\right)\left(\Pi_{p,x}^{\delta }{}^{2}+a_{23}{\left(\frac{t}{D_{b}}\right)}_{eff}\right)\left(\Pi_{p,x}^{\delta }{}^{2}+a_{24}{\left(\frac{t}{D_{b}}\right)}_{eff}\right).$$

(A8) $$B_{1}=b_{01}+b_{02}\;n+b_{03}{\left(\frac{t}{D_{b}}\right)}_{eff}+b_{04}\;n{\left(\frac{t}{D_{b}}\right)}_{eff}+n{\left(\frac{t}{D_{b}}\right)}_{eff}\left(b_{05}n+b_{06}\;{\left(\frac{t}{D_{b}}\right)}_{eff}\right)+b_{07}{\left(\frac{t}{D_{b}}\right)}_{eff}^{2}.$$

(A9) $$B_{2}=b_{08}+b_{09}\;\Pi_{V}^{\delta }+{\left(\frac{t}{D_{b}}\right)}_{eff}\left(b_{10}+b_{11}\;n^{3}\right)+{\left(\frac{t}{D_{b}}\right)}_{eff}^{2}\left(b_{12}+b_{13}\;n^{2}\right).$$

(A10) $$B_{3}=b_{14}+b_{15}\;n+b_{16}\;\Pi_{V}^{\delta }+b_{17}\;{\left(\Pi_{V}^{\delta }\right)}^{2}+\frac{1}{b_{18}+b_{19}\;{\left(\Pi_{V}^{\delta }\right)}^{2}}+b_{20}{\left(\frac{t}{D_{b}}\right)}_{eff}.$$

(A11) $$B_{4}=b_{21}\;sin\left(b_{22}\;\Pi_{V}^{\delta }\right).$$

(A12) $$B_{5}=b_{23}+b_{24}\;\Pi_{V}^{\delta }+b_{25}\;{\left(\Pi_{V}^{\delta }\right)}^{2}+b_{26}{\left(\frac{t}{D_{b}}\right)}_{eff}.$$

(A13) $$B_{6}=n\left(b_{27}+\frac{b_{28}}{b_{29}-{\left(\Pi_{V}^{\delta }\right)}^{2}}+b_{30}{\left(\frac{t}{D_{b}}\right)}_{eff}\right).$$

### Appendix A.2. Model Coefficients

**Table A1:** Model coefficients.

$a_{00}$	0.0000413479	$b_{00}$	0.998487
$a_{01}$	−2.97497	$b_{01}$	−11.4595
$a_{02}$	390.644	$b_{02}$	9.75142
$a_{03}$	−1.00056	$b_{03}$	1.69836
$a_{04}$	−0.121986	$b_{04}$	−0.775636
$a_{05}$	−0.394801	$b_{05}$	−0.533892
$a_{06}$	−0.317506	$b_{06}$	0.1505
$a_{07}$	−0.093065	$b_{07}$	0.063818
$a_{08}$	0.762303	$b_{08}$	0.910203
$a_{09}$	0.11879	$b_{09}$	−0.148087
$a_{10}$	0.121586	$b_{10}$	0.169683
$a_{11}$	4.04094	$b_{11}$	−0.471171
$a_{12}$	11.6959	$b_{12}$	0.015469
$a_{13}$	89.2206	$b_{13}$	0.30448
$a_{14}$	−0.147822	$b_{14}$	1.74533
$a_{15}$	0.0113796	$b_{15}$	0.343839
$a_{16}$	−0.0381859	$b_{16}$	−3.1969
$a_{17}$	0.0171605	$b_{17}$	1.597370
$a_{18}$	791.442	$b_{18}$	−67815.8
$a_{19}$	−1.01937	$b_{19}$	67789.9
$a_{20}$	0.209565	$b_{20}$	0.00156
$a_{21}$	4118.38	$b_{21}$	1.01936
$a_{22}$	−0.26066	$b_{22}$	1.45018
$a_{23}$	0.00593881	$b_{23}$	17.2059
$a_{24}$	0.26419	$b_{24}$	−13.3529
		$b_{25}$	6.68431
		$b_{26}$	0.35355
		$b_{27}$	−2.15945
		$b_{28}$	−0.00005
		$b_{29}$	0.99991
		$b_{30}$	−0.368

**Table A2:** Nomenclature.

$a_{ij}$	coefficients of regression model	$\dot{V}$	volume flow rate
$A_{i}$	subfunctions of regression model	w	channel width
$AE_{max}$	maximum absolute error	x	cross-channel coordinate
$AE_{mean}$	mean absolute error	x	vector of unknowns
$b_{ij}$	coefficients of regression model	y	up-channel coordinate
$B_{i}$	subfunctions of regression model	$y_{i}$	numerical result
$C_{1,2}$	constants	${\hat{y}}_{i}$	approximated result
$D_{b}$	barrel diameter	$\bar{y}$	mean value of numerical results
D	rate-of-deformation tensor	z	down-channel coordinate
e	flight width	α	temperature coefficient
f	vector of boundary conditions	δ	flight clearance
i	number of parallel screw flights	$\dot{\gamma }$	shear rate
J	Jacobian	${\dot{\gamma }}_{\delta }^{*}$	dimensionless shear rate
K	consistency	η	viscosity
L	velocity gradient tensor	$\eta_{\delta }^{*}$	dimensionless viscosity
$\dot{m}$	mass-flow rate	$v_{i}$	dimensionless velocities
n	power-law index	ξ	dimensionless height direction
N	screw speed	$\Pi_{p,i}^{\delta }$	dimensionless pressure gradients
$R^{2}$	coefficient of correlation	$\Pi_{Q}^{\delta }$	dimensionless dissipation
$RE_{max}$	maximum relative error	$\Pi_{Q,app}^{\delta }$	approximated dimensionless dissipation
$RE_{mean}$	mean relative error	$\Delta \Pi_{Q}^{\delta }$	corrected dimensionless dissipation
p	pressure	$\Pi_{V}^{\delta }$	dimensionless flow rate
t	screw pitch	$\Pi_{V,app}^{\delta }$	approximated dimensionless flow rate
$v_{i}$	velocities	$\Delta \Pi_{V}^{\delta }$	corrected dimensionless flow rate
$v_{b}$	barrel velocity	$\rho_{m}$	melt density
$v_{b,x}$	barrel velocity in x-direction	$\tau_{ij}$	shear stresses
$v_{b,z}$	barrel velocity in z-direction	τ	stress tensor
v	velocity vector	$\varphi_{b}$	pitch angle
